# Association of sleep quality with insulin resistance in obese or overweight subjects

**DOI:** 10.5935/1984-0063.20200084

**Published:** 2021

**Authors:** Sima Hashemipour, Azam Ghorbani, Atoosa Khashayar, Hamideh Olfati

**Affiliations:** 1 Associate Professor of Endocrinology. Metabolic Diseases Research Center, Research Institute for Prevention of Non-Communicable Diseases, Qazvin University of Medical Sciences, Qazvin, Iran.; 2 Instructor of Nursing, Metabolic Diseases Research Center, Research Institute for Prevention of Non-Communicable Diseases, Qazvin University of Medical Sciences, Qazvin, Iran.; 3 General Practitioner, Metabolic Diseases Research Center, Research Institute for Prevention of Non-Communicable Diseases, Qazvin University of Medical Sciences, Qazvin, Iran.; 4 Internist, Metabolic Diseases Research Center, Research Institute for Prevention of Non-Communicable Diseases, Qazvin University of Medical Sciences, Qazvin, Iran.

**Keywords:** Insulin Resistance, Sleep Quality, Obesity, Overweight

## Abstract

**Introduction:**

Obesity or overweight are two factors associated with insulin resistance (IR). There are limited studies with regard to the role of some non-traditional factors such as sleep quality in level of IR in obese individuals. The current study aimed at investigating the association of sleep quality with IR in overweight or obese people.

**Material and Methods:**

In this cross-sectional study, 612 obese or overweight participants of the Qazvin metabolic disease study (QMDS) were evaluated. Sleep quality was measured using the Pittsburgh sleep quality index (PSQI) and compared between two groups of participants with and without IR.

**Results:**

Our findings showed that the total score of sleep quality in the IR group was significantly lower than that in the non-insulin resistant group (8.78±2.78 vs. 8.13±2.70, p=0.008). After adjustment, each unit increase of the sleep latency and subjective sleep quality scores was associated with a 1.23 and 1.33 times increased risk of IR, respectively (p<0.05).

**Conclusion:**

In the obese or overweight people, sleep quality is associated with IR.

## INTRODUCTION

In recent decades, the relationship between sleep disorders and insulin resistance (IR) has been considered as a remarkable research topic. Many studies have reported the association of sleep quality and sleep pattern with increased risk of obesity, hypertension, cardiovascular diseases^1^ and insulin resistance^2^. Short-term sleep restriction (even one to three nights) can lead to insulin resistance in healthy people through several metabolic pathways^3,4^. Sleep restriction in healthy people leads to a decrease (approximately 30%) in cellular insulin signaling and IR in fat cells^5^. On the other hand, long sleep duration (>7h) can cause obesity and IR^6^. For many years, the word “obesity” has been connected with IR. However, IR and the metabolic syndrome are not common in all obese or overweight people. The contributing factors for such difference in individuals with obesity are not completely figured out, and the results of studies investigating the role of some potential factors such as poor sleep quality and sleep duration are inconsistent^7,8,9^. In some of these studies, there was no relationship between total score of sleep quality and sleep duration with metabolic syndrome in obese people^7,8^. Nevertheless, there are reports indicating the association between shorter duration of sleep and the metabolic syndrome in obese women, as compared to their healthy obese counterparts^9^. In addition, the relationship between metabolic syndrome (except IR) and sleep quality in obese subjects have been investigated in previous studies.

Due to the contradiction of the reported results and limited data concerning the role of sleep quality in IR in obese individuals, this study was designed to evaluate the relationship between sleep quality and quantity with IR in overweight and obese people.

## MATERIAL AND METHODS

This cross-sectional study was carried out on obese or overweight residents of Minoodar, Qazvin, Iran, participating in Qazvin metabolic disease study (QMDS). The study was approved by the Ethics Committee of the Qazvin University of Medical Sciences. Sampling was performed using the multistage cluster random sampling technique. Inclusion criteria were the age of 20 or above and BMI of 25 or above. Subjects with end-stage liver or kidney disease, non-cured cancer, and pregnant women were excluded from the study. The participants were invited via phone and the details and aims of the study were explained to them. The individuals were free to participate in the study and all of them signed an informed consent form. A questionnaire on clinical examination and medical history of the participants was filled out by two general practitioners. The details of sampling and data collection have already been published elsewhere^10^.

IR was calculated using the formula: HOMA-IR=fasting blood sugar (mmol/lit) ×insulin (micro unit/ lit)/22.5^11^.

The 75^th^ percentile of HOMA-IR in subjects without metabolic syndrome of our study population was use as the cutoff for defining of insulin resistance. This value was 3.42. So, cut-off of 3.4 was used for definition of insulin resistance in our overweight/obese population study^12^.

Sleep quality was examined through self-report and by using the Pittsburgh sleep quality index (PSQI). The PSQI consisted of 19 items. In this questionnaire, higher scores of the PSQI show poorer sleep quality^13^. The collected data were analyzed in the SPSS software v. 24. The chi-square test and t-test test were used to compare gender distribution and age, sleep related parameters between the insulin and non-insulin resistant groups, respectively. The logistic regression analysis was used to investigate whether there was an independent relationship between the sleep-related factor and insulin resistance after adjustment for gender, age, and BMI.

## RESULTS

Results of 612 participants were recorded. In total, 223 participants (36.4%) had IR and participants with IR had higher BMI (29.63±3.36 compared to 28.33±2.72, *p*<0.001). Sleep quality was lower in the IR group compared to non-IR group (8.78±2.78 and 8.13±2.70 in insulin, respectively, *p*=005) (Table 1).

**Table 1:** Comparison of demographic and sleep quality components between insulin resistance and non-insulin resistance groups.

	Non- insulin resistance group N=389	Insulin Resistance group N=223	P-value
Age (year)^a^	42.07±8.27	43.65±8.55	0.026
Gender (% of female)	229(58.4)	122(54.5)	0.353
BMI (kg/m^2^)	28.40±3.00	29.61±3.31	<0.001
Subjective sleep quality	0.95±0.64	1.07±0.60	0.032
Sleep latency	1.01±0.91	1.18±0.88	0.021
Sleep duration score	0.83±0.77	0.85±0.79	0.754
Habitual sleep efficiency	2.61±0.85	2.74±0.72	0.071
Sleep disturbances	1.16±0.49	1.21±0.57	0.207
Use of sleep medication	0.21±0.62	0.30±0.76	0.114
Daytime dysfunction	1.33±0.81	1.35±0.82	0.703
Total PSQI*	8.13±2.70	8.73±2.76	0.008

Data are presented as mean plus and minus standard deviation except those pointed in the table as percent.

* Pittsburgh sleep quality index

As presented in Table 1, the scores of subjective sleep quality and sleep latency in the insulin resistance group were significantly worse than the non-IR group. The sleep duration score was not significantly different between the two groups.

For each unit increase of the total sleep quality score, there was a 1.08 times elevated risk of IR (CI: 1.02-1.15, *p*=0.009). Moreover, each unit increase of the sleep latency and subjective sleep quality scores was associated with a 1.25 and 1.34 times increased risk of insulin resistance, respectively (*p*=0.016 and *p*=0.027, respectively). After adjusting for age, gender and BMI, the relationship between total sleep quality score, sleep latency and subjective sleep quality with insulin resistance still remained significant (*p*=0.019, *p*=0.027 and *p*=0.039, respectively). The sleep duration and time to go to bed were not associated with insulin resistance (Table 2).

**Table 2:** Logistic regression analysis of the relationship between sleep related factors and insulin resistance

Variable		Crude OR	P-value	OR*	P-value
**PSQI Score**		1.084 (1.020-1.151)	0.009	1.08 (1.013-1.15)	0.018
	Subjective sleep quality	(1.023-1.722) 1.237	0.033	(1.015- 1.754) 1.242	0.039
	Sleep latency	(1.032-1.483)	0. 022	(1.025- 1.506) 1.025- 1.506)	0.027
	Sleep duration score	1.034 (0.838-1.277)	0. 754	(0.854- 1.324)	0.582
**PSQI factors**	Habitual sleep efficiency	1.220 (0.981-1.516)	0. 073	1.210 (0.970- 1.51)	0.091
	Sleep disturbances	1.222 (0.895-1.667)	0. 207	1.104 (0.796- 1.531)	0.554
	Use of sleep medication	1.208 (0.851-1.270)	0.116	1.163 (0.902- 1.487)	0.230
	Daytime dysfunction	1.040 (0.851-1.270)	0. 703	1.027 (0.835- 1.265)	0.798
**Wake up time**	6-7 am	1		1	
	<6 am	0.719 (0.424-1.220)	0.222	0.697] (0.401-1.209)	0.199
	>7 am	0.785 (0.543-1.133)	0.196	0.830 (0.564- 1.222)	0.345
	6-8 hour	1		1	
	<6 hour	1025 (0.434-2.421)	0. 956	0.834 (0.328- 2.122)	0.704
**Sleep duration**	>8 hour	0.670 (0.450-0.999)	0. 049	0.673 (0.442- 1.023)	0.064

## DISCUSSION

In this study, worse sleep quality was associated with insulin resistance in the obese or overweight people. The major difference in sleep quality was related to sleep latency and subjective sleep quality in the insulin resistance group. Sleep quality, sleep latency, and subjective sleep quality were the independent predictors for the risk of insulin resistance in the obese people.

The association between poor sleep quality and quantity with metabolic disorders has been reported in previous studies^14^.

The present study is one of the few population-based studies conducted on overweight/obese people for investigating the relationship between sleep quality and duration with IR. In a cross-sectional study by Kanagasaba et al. (2017)^7^, which was conducted on 1,777 obese people in the United States, neither sleep duration nor the total score of sleep quality was associated with metabolic disorder. Nevertheless, the components of sleep quality such as sleep latency, frequent waking up during the night, feeling sleepy, as well as feeling restless during the day were associated with higher risk of metabolic disorder^7^. Despite Hankinson et al. (2013)^9^ study, we did not find any association of sleep duration and IR. In the study by Gonzaga et al. (2016)^15^ which was conducted on overweight or obese children, among metabolic syndrome components, only high blood pressure was associated with sleep quality.

The reasons of such differences in reported results are not clear. Some factors such as race^15,16^ and psychological factors^17,18^ may affect the relationship of sleep quality and IR.

In the study of Jennings et al. (2007)^17^, sleep quality was associated with metabolic syndrome. However, after adjustment of depression, there was no association between sleep quality and metabolic syndrome. In the study buy Nguyen-Rodriguez et al. (2010)^18^, high sleep latency was associated with emotional eating. The results of multivariate models showed that trait anxiety was an essential factor for emotional eating in people with high sleep latency.

Low sleep quality is associated with changes in appetiteregulating hormones, increased sympathetic tone and higher cortisol secretion, which can lead to increased insulin resistance^19^. However, the relationship between sleep quality and insulin resistance may be bidirectional. In a prospective study by Balkau et al. (2010)^20^, the HOMA-IR value was a predictive factor for the incidence of obstructive sleep apnea during 6 years.

This study had some limitations. The cross-sectional design, subjective assessment of sleep quality, and not evaluating other sleep problems such as obstructive apnea were the main limitations of this study.

In conclusion, in our study, IR in the obese or overweight subjects was associated with lower quality of sleep mainly due to increased sleep latency and worse subjective sleep quality. For investigating causal relationship of insulin resistance and sleep quality in obese subjects, prospective studies are necessary.
